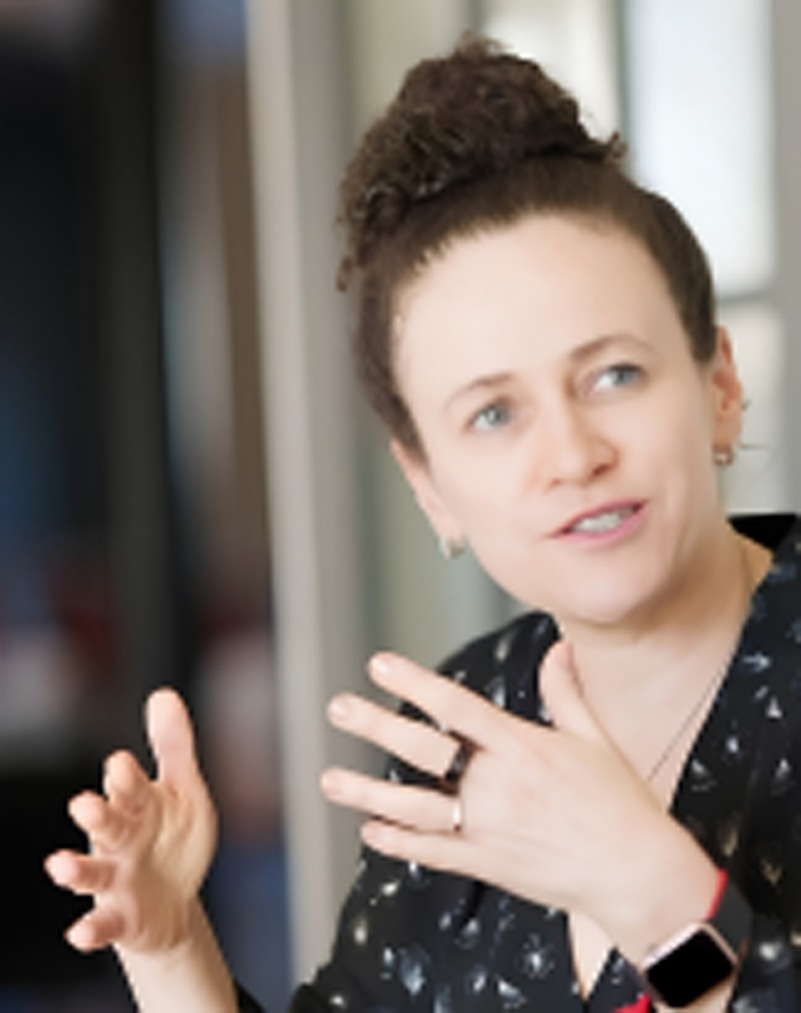# The 2026 ISCB Innovator Award—Dr Olga Troyanskaya

**DOI:** 10.1093/bioinformatics/btag281

**Published:** 2026-07-07

**Authors:** Mallory L Wiper

**Affiliations:** The International Society for Computational Biology, 525K East Market Street, RM 330, Leesburg, VA 20176, United States


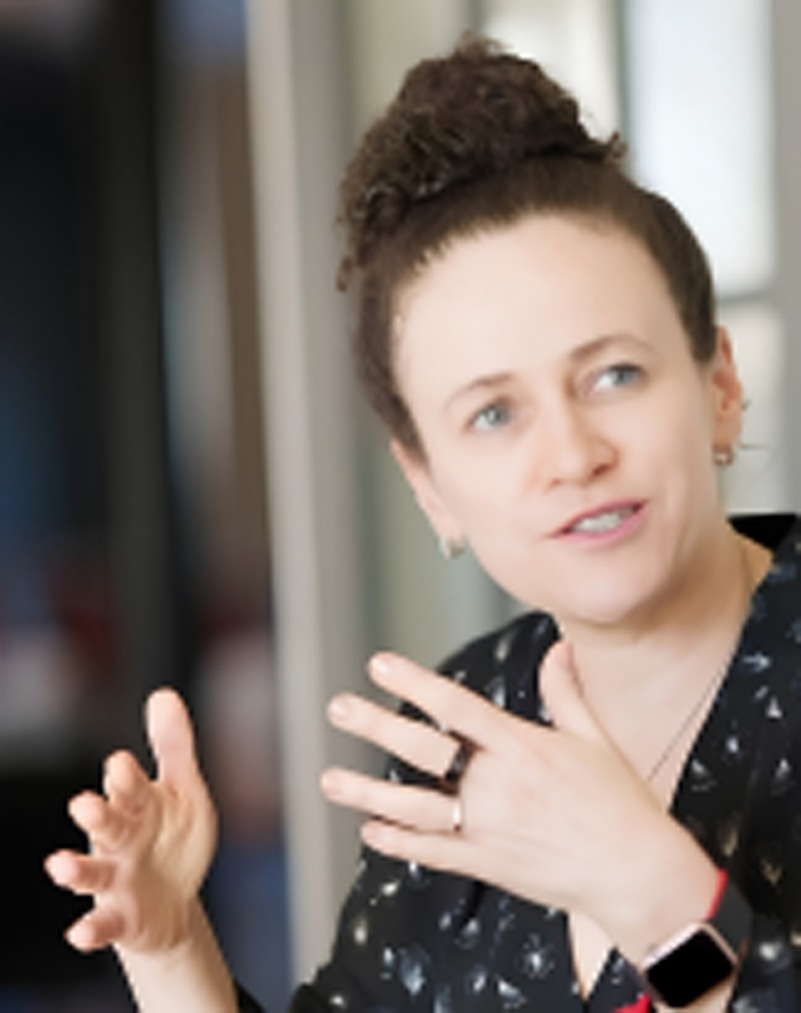


The International Society for Computational Biology (ISCB) is delighted to present the 2026 ISCB Innovator Award to Dr Olga Troyanskaya in recognition of her innovative work developing computational and machine learning approaches to interpret complex biological data and advance precision health.

## 1 Early curiosity and discovery

Dr Troyanskaya recalls always being interested in math and science, and from a young age was very curious about the world—so much so that she remembers being crushed when she realized that humans would never have all the answers to how the world works. In middle school, she recalls finding a book of her mother’s about Mendelian genetics and chromosomal abnormalities that immediately captivated her, shifting her perspective on the limits of scientific knowledge. Rather than a source of discouragement, those limits became part of the mystery of the world.

The question of where to direct her curiosity truly took shape for Troyanskaya during her undergraduate years. She had planned to triple major in economics, math, and computer science at the University of Richmond but quickly became uninterested in economics. She still harbored her fascination with biology, though—especially with molecular biology and genetics—and was certain there had to be ‘something’ at the intersection of computer science and biology. So, she started searching.

Troyanskaya recalls going through every major university on the East Coast that had a top computer science department and a top biology department and emailing faculty asking about research opportunities. Only one person responded: Steven Salzberg, then at Johns Hopkins. That one email led to a summer opportunity to work in Salzberg’s lab, introducing her to computational biology at an exciting time in the field, when large-scale genomic sequencing efforts were starting to take shape. She calls this experience one of ‘many’ serendipitous moments in her life. After her summer in Salzberg’s lab, she returned to Richmond and dropped economics, declaring a double major in computer science and biology.

## 2 Influential mentors

Troyanskaya’s academic career was shaped by three major mentors: Steven Salzberg, David Botstein, and Russ Altman.

Salzberg was instrumental in exposing Troyanskaya to computational biology and sequence analysis. He opened an entire scientific world that Troyanskaya is certain she wouldn’t have discovered without him. In addition to opening the door to computational biology, Salzberg provided an unforgettable example of what it means to be a mentor that truly supports and nurtures curiosity.

By graduate school, Troyanskaya had set her sights on understanding genome-scale processes happening in the cell. To explore this topic thoroughly, she chose to attend Stanford University, not only because she had heard about the emerging technology of microarrays being worked on at Stanford but also because she wanted to work with Botstein and Altman.

Working with Botstein gave Troyanskaya a computational mentor who pushed her to think more deeply about biology and genetics. From Altman, she learned about structure while also gaining insights into computational analysis and how to frame complex problems.

She credits her growth and development as a scientist to her graduate school years learning from Botstein and Altman. Both taught her how to communicate effectively as a scientist, but more importantly, they helped Troyanskaya develop what she calls one of the most critical scientific skills: learning how to ask the right questions.

## 3 Fostering scientific independence

A key lesson Troyanskaya took from her mentors, and which she implements in her own lab, is that mentorship should not be uniform; different students need different types of support. Some students may need a lot more direct support in the beginning, while others are happy with more freedom and less guidance. She emphasized that strong mentorship comes from understanding your students’ goals, strengths, and what they need to learn most. That information can help inform how each person needs to be mentored.

Taking the time to understand students in this way is part of creating an environment where students feel comfortable asking questions, sharing opinions, and presenting ideas. An environment encouraging the open exchange of ideas was something she saw modeled most clearly by Botstein. Despite being known for his strong personality, Botstein always welcomed rigorous scientific debate within his lab. Students knew that they could disagree with him, and especially if they presented a cogent argument, he was happy to be wrong. This openness to discussion and new ideas is something Troyanskaya tries to emulate in her own lab and mentoring practices.

## 4 Expanding scientific inquiry

A central part of Troyanskaya’s research philosophy comes back to what she learned from her mentors: identifying the right questions. For her lab, this means asking not just whether a problem is worth solving but whether they are uniquely placed to solve it. This involves considering whether their integration of data analysis and computational methods can make a contribution that is unique or genuinely impactful.

As the PI of a technology lab deeply rooted in biomedical and health research, Troyanskaya can ask questions on a larger and more diverse scale than when she was in graduate school, with her pursuits now including areas such as neurodegeneration, kidney disease, and autism. Central to this exploration of varied questions is collaboration with domain experts. In this way, the lab stays grounded in its expertise in biology rather than, as Troyanskaya puts it, building spherical cows.

## 5 Unexpected discoveries and new directions

One of the most out-of-the-box findings in Troyanskaya’s lab was the result of work by senior researcher Natalie Sauerwald, who looked at phenotypic data from the Simons Foundation SPARK cohort to determine whether autism phenotypes could be clustered into subgroups. Much to Troyanskaya’s surprise, Sauerwald’s analysis ‘did’ identify remarkably distinct phenotypic subgroups of autism. This exciting result revealed something that was as yet unknown, offering new insights and meaningfully impacting future research.

Technological opportunities and advances have also shifted the focus of Troyanskaya’s research. For instance, though she hadn’t expected it, her group moved into using deep learning models, leading to the development of DeepSEA to predict epigenetic signals from DNA sequences.

Troyanskaya is currently most interested in the interdisciplinary initiative she spearheads at Princeton: Princeton Precision Health. This initiative aims to understand human health at a much deeper and more integrated level. With a deeper understanding of human health, Troyanskaya hopes to develop integrative computational models capable of understanding health at a population, individual, and mechanistic molecular level.

Answering these big questions continues to fuel Troyanskaya’s passion for discovery about the world because, in her words, “Science is an exciting adventure!”

## 6 Reflections on the Innovator Award

Troyanskaya said she’s deeply honored to be named this year’s winner while emphasizing that this isn’t an achievement she’s reached on her own. For her, the award reflects the efforts of her lab’s trainees and senior researchers, as well as collaborators and mentors, particularly David Botstein. Botstein’s impact on her career is something Troyanskaya is extraordinarily grateful for. In tribute to her mentor, she dedicates this award to him.